# Tumoral cubilin is a predictive marker for treatment of renal cancer patients with sunitinib and sorafenib

**DOI:** 10.1007/s00432-017-2365-y

**Published:** 2017-03-04

**Authors:** Marjut Niinivirta, Gunilla Enblad, Per-Henrik Edqvist, Fredrik Pontén, Anca Dragomir, Gustav J. Ullenhag

**Affiliations:** 1 Department of Immunology, Genetics and Pathology, Uppsala University, Uppsala, Sweden; 2 Department of Oncology, Uppsala University Hospital, Entrance 78, 751 85 Uppsala, Sweden; 3 Department of Immunology, Genetics and Pathology and Science for Life Laboratory, Uppsala University, Dag Hammarskjölds väg 20, 751 85 Uppsala, Sweden; 4 Department of Surgical Pathology, Uppsala University Hospital, 75185 Uppsala, Sweden

**Keywords:** Cubilin, Predictive marker, Renal cancer, Tissue microarray, Tyrosine kinase inhibitor

## Abstract

**Purpose:**

Tyrosine kinase inhibitors like sunitinib and sorafenib are commonly used to treat metastatic renal cell cancer patients. Cubilin is a membrane protein expressed in the proximal renal tubule. Cubilin and megalin function together as endocytic receptors mediating uptake of many proteins. There is no established predictive marker for metastatic renal cell cancer patients and the purpose of the present study was to assess if cubilin can predict response to treatment with tyrosine kinase inhibitors.

**Methods:**

Cubilin protein expression was analyzsed in tumor tissue from a cohort of patients with metastatic renal cell cancer (*n* = 139) using immunohistochemistry. One hundred and thirty six of the patients were treated with sunitinib or sorafenib in the first- or second-line setting. Thirty of these were censored because of toxicity leading to the termination of treatment and the remaining (*n* = 106) were selected for the current study.

**Results:**

Fifty-three (50%) of the tumors expressed cubilin in the membrane. The median progression-free survival was 8 months in patients with cubilin expressing tumors and 4 months in the cubilin negative group. In addition, the overall survival was better for patients with cubilin positive tumors. We also found that the fraction of cubilin negative patients was significantly higher in the non-responding group (PFS ≤3 months) compared to responding patients (PFS >3 months).

**Conclusions:**

We show for the first time that tumoral expression of cubilin is a positive predictive marker for treatment of metastatic renal cell cancer patients with sunitinib and sorafenib.

## Introduction

Renal cell carcinoma (RCC) is highly resistant to chemotherapy and radiotherapy (Motzer et al. 1996). Cytokine therapy (high-dose interleukin 2 (IL-2) and interferon-alpha (IFN-*α*) has been approved for the treatment of metastatic renal cell carcinoma (mRCC). IFN-α is the most frequently used cytokine and it achieves an objective response rate of 7.5% and a median overall survival (OS) time of 13 months (Negrier et al. 1998).

The introduction of targeted therapy has changed the standard of treatment for mRCC. Tyrosine kinase inhibitors (TKI); sunitinib, sorafenib, pazopanib and axitinib, act by blocking essential biochemical pathways or proteins that are required for tumor cell growth and survival (Cho and Chung 2012).

Sunitinib and sorafenib, two common used TKIs, are the focus for this study. Both these agents target the receptors of vascular endothelial growth factor (VEGF) and platelet-derived growth factor (PDGF) (Escudier et al. 2012). In the first-line setting in metastatic disease, the median progression-free survival (PFS) extends to 11 months for sunitinib and in the second line setting to 5.5 months for sorafenib in selected patients (Escudier et al. 2007; Motzer and A 2007).

Several prognostic factors are established for RCC patients like number of metastatic sites, time from diagnosis to treatment, Karnofsky performance status, hemoglobin, white blood count, platelets count, lactate dehydrogenase, alkaline phosphatase and “corrected” serum calcium (Heng et al. 2009; Manola et al. 2011). Though, considering possible severe toxicity and the costs of TKIs there is a need of predictive factors to select which patients will gain from the treatment.

The majority of studies predicting effects of sunitinib treatment in mRCC patients are based on serum proteins. Tumor necrosis factor α (TNFα) and metalloproteinase-9 (MMP-9) baseline levels were significantly increased in non-responders and significantly associated with reduced OS and time-to-progression (Perez-Gracia et al. 2009). In a study of circulating, VEGF and neutrophil gelatinase-associated lipocalin (NGAL) pre-treatment levels were significant predictors of PFS (Porta et al. 2010). Tumoral molecular markers, such as HIF-1-α, CA9, Ki67, CD31, pVEGFR1, VEGFR1 and VEGFR2, pPDGFR-α and -β might predict a good response to sunitinib treatment (Dornbusch et al. 2013). Developing hypertension, a well-known side effect of sunitinib-therapy, was associated with significantly longer OS and PFS in a study of 111 patients with mRCC (Szmit et al. 2012a).

Hypertension induced by sorafenib is also a factor for early response evaluation (Szmit et al. 2012b).

Fewer predictive markers studies have been published for sorafenib than for sunitinib. In one study, patients with higher baseline plasma levels of VEGF benefitted more from sorafenib in terms of PFS than those with low levels (Escudier et al. 2009). In another study, levels of circulating cell-free DNA (cfDNA) had in a small subset of mRCC patients no predictive value at baseline but during sorafenib treatment (Feng et al. 2013).

Sorafenib is also used to treat hepatocellular cancer (HCC) (Llovet et al. 2008). Nine serum cytokines (angiopoietin-2 (Ang-2), follistatin, granulocyte colony stimulating factor (G-CSF), hepatocyte growth factor (HGF), interleukin-8 (IL-8), leptin, PDGF-BB, platelet endothelial cell adhesion molecule-1 and VEGF) were measured in 30 HCC-patients treated with sorafenib. PFS was significantly shorter in patients with high levels of Ang-2, G-CSF, HGF and leptin at baseline (Miyahara et al. 2011).

Cubilin (CUBN) is a high molecular weight endocytic receptor expressed in proximal renal tubule (Christensen and Verroust 2002). It is a membrane protein which interacts with megalin, another endocytic receptor, in the proximal tubule for effective reabsorption of filtered proteins including albumin, transferrin, vitamin D-binding protein and other important plasma carriers (Christensen et al. 2013).

Cubilin is also found in several other epithelia including the visceral yolk sac and to a lesser extent the ileal and the uterine mucosa (Verroust and Kozyraki 2001).

While cubilin has no known function in cancer, it was identified as a potentially interesting protein through systematic researches within The Human Protein Atlas (http://www.proteinatlas.org) internal database for proteins. It was selected for further studies based on highly specific expression patterns in normal kidney and renal cancers on both immunohistochemistry level and RNA level (Ponten et al. 2011). We have recently demonstrated that expression of cubilin is highly specific for RCC and that loss of cubilin expression is associated with poor prognosis (Gremel et al. 2017).

The aim with the present study was to explore the potential value of tumoral expression of cubilin as a predictive marker for TKI treatment in mRCC patients.

## Materials and methods

### Patients

In an attempt to overcome some of the reporting deficiencies inherent in tumor marker studies, we followed the REporting recommendations for tumor MARKer studies (REMARK) (McShane et al. 2005) when compiling this manuscript.

The cohort consisted of 139 patients in seven Departments of Oncology in Sweden: Uppsala (*n* = 48), Göteborg (*n* = 36), Örebro (*n* = 19), Västerås (*n* = 12), Gävle (*n* = 11), Falun (*n* = 7) and Karlstad (*n* = 6). These patients were diagnosed with mRCC between 2006 and 2010. All the patients had a prior nephrectomy and were thereafter treated with various therapeutic agents: TKIs (sunitinib and sorafenib), mTOR inhibitor (temsirolimus), IFN-α and/or bevacizumab. The patients (*n* = 136) treated with sunitinib (registered dosing is 50 mg daily for 4 weeks followed by 2 weeks rest) or sorafenib (registered dosing is 400 mg two times daily continuosly) in the first- or second-line setting were selected for the current study. Twenty of these had been treated with IFN-*α* before receiving a TKI.

Clinical data was collected, including the patient’s age, gender and histologic subtype (Table 1) as well as the length of treatment with sunitinib and sorafenib. Progression-free survival was calculated as the time from the start of treatment to the time of clinical and/or radiological progression, treatment discontinuation due to toxicity or end of follow-up. We also registered the OS calculated from the diagnosis of mRCC. We defined the patients experiencing a PFS of ≤3 months as the non-responding group.

**Table 1:** Clinical characteristics of renal cancer patients treated for metastatic disease with sunitinib or sorafenib in the first- or second-line setting

Patient cohort	Total *n* = 106
Gender, *n* (%)	
Male	77 (73)
Female	29 (27)
Age at diagnosis, years	
Median (range)	62.5 (33–77)
Age at metastatic disease, years	
Median (range)	65 (34–84)
Histologic type, *n* (%)	
Clear cell	89 (84)
Papillary	4 (4)
Mixed phenotype	4 (4)
Unknown	9 (8)
Local disease at diagnosis, *n* (%)	49 (46)
Metastatic disease at diagnosis, *n* (%)	57 (54)
Time to metastasis, years	
Median (range)	2 (0–18)
Metastasis during first year, *n* (%)	20 (41)
Metastasis after first year, *n* (%)	29 (59)
Alive, *n* (%)	17 (16)
Dead, *n* (%)	89 (84)

### Tissue microarray (TMA) generation

TMA, immunohistochemistry and slide scanning were essentially performed in accordance to standards used in the Human Protein Atlas (http://www.proteinatlas.org) (Kampf et al. 2012; Ponten et al. 2011). In brief, corresponding HE slides were examined and representative regions from the primary tumors selected for the TMA. For each patient, two cores (1 mm in diameter) containing tumor tissue were collected (except in one case where there was only enough material for one core) by punch biopsy and transferred to recipient paraffin blocks subsequently containing 277 cores. TMArrayer™ (Pathology Devices, Westminster, MD, USA) and the Beecher Instruments Manual Tissue Arrayer MTA-1 (Estigen OÜ, Tartu, Estonia) were used for this procedure.

### Immunohistochemical methods

Immunohistochemistry and slide scanning was performed at the Swedish Science for Life Laboratory (SciLifeLab) facilities in the Department of Immunology, Genetics, and Pathology at the Rudbeck Laboratory of Uppsala University. In brief, 4-μm TMA sections collected on SuperFrost Plus slides were prior to immunostaining deparaffinised in xylene, re-hydrated in graded alcohols, blocked for endogenous peroxidase, and subjected to heat-induced antigen retrieval. Automated IHC was performed using a LabVisionAutostainer 480S (Thermo Fisher Scientific, Runcorn, UK). Primary antibody towards cubilin (HPA004133, Atlas Antibodies, Stockholm Sweden) was validated for immunohistochemistry according to established criteria (Kampf et al. 2012). The antibody was diluted 1:125 in UltraAb Diluent (Thermo Fisher Scientific, Fremont, CA, USA) and applied to the slides for 30 min at room temperature. The slides were further incubated with the secondary reagent, an anti-rabbitmouse horse reddish peroxidase-conjugated UltraVision (Thermo Fisher Scientific, Runcorn, UK) for 30 min at room temperature. Following the washing steps, the slides were developed for 10 min using the avidin–biotin peroxidase staining technique (Vector elite; Vector Laboratories, Burlingame, CA, USA), using 3.3-diaminobenzidine as the substrate. The slides were then counterstained with Mayer’s haematoxylin for 5 min (Sigma–Aldrich, St.Louis, MO, USA) and coverslipped with Pertex (HistolabAB, Gothenburg, Sweden).

### Slide scanning and evaluation of staining

To obtain high-resolution digital images, the IHC slides were scanned with a ×20 objective using the AperioScanScope XT Slide Scanner (Aperio Technologies, Vista, CA, USA).

The digital images were examined in duplicates on a colour-calibrated screen using ImageScope (Aperio, Vista, CA, USA). Staining in the live tumor cells was semi-quantitatively evaluated by two observers, of which one pathology specialist (MN and AD) and disagreements were resolved by re-evaluation of the images. MN and AD did not know the patient’s information until they completed evaluation of staining. Two cellular compartments were annotated: cytoplasm and membrane. For the membrane staining, both intensity (circumference) and fraction of stained cells were categorically estimated using a scale of 0–2 for the intensity (0 = negative = 0–10% of the cells circumference stained, 1 = incomplete = 11–80% of the cells stained, 2 = complete = 81–100% of the cells circumference stained), respectively, 0–4 for the fraction (0 = 0–1%, 1 = 2–25%, 2 = 26–50%, 3 = 51–75%, 4 = 76–100%).

The distribution of the results for the membranous expression is given in Table 2.

**Table 2:** Distribution of membrane staining results among primary tumors of renal cancer from patients treated for metastatic disease with sunitinib or sorafenib in first- or second-line setting

Intensity score	Percentage stained (%)	Number of cases
Intensity (circumference) of stained membrane and distribution		
0	0–10	46
1	11–80	26
2	81–100	34

Fraction score	Percentage stained (%)	Number of cases
Fraction of stained membrane and distribution		
0	0–1	55
1	2–25	20
2	26–50	21
3	51–75	5
4	76–100	5

Combined score	Number of cases
Combined score (addition of intensity and fraction score) and distribution	
0	46
1	7
2	10
3	19
4	17
5	3
6	4

Cubilin expression	Number of cases
Cubilin negative and positive cases	
(−)	53
(+)	53

**Fig. 1:**
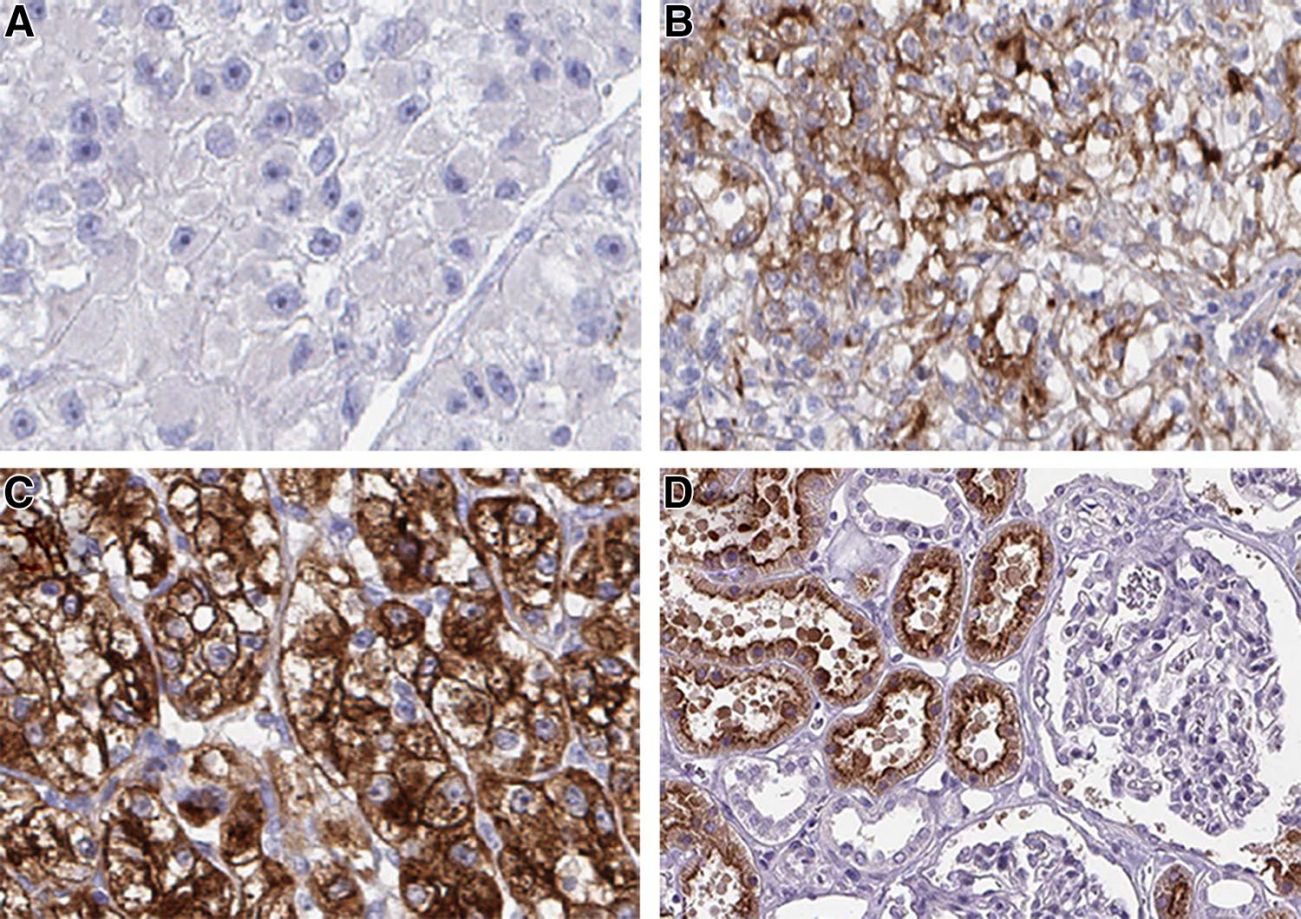
Representative images of the immunohistochemistry results for cubilin from primary renal cell carcinomas, from patients later treated for metastatic disease with sunitinib or sorafenib in the first- or second-line setting, demonstrating negative (**a**), incomplete (**b**) and complete (c) membranous staining in tumor cells and normal kidney (**d**). Magnification ×200

Representative examples of negative, incomplete and complete membranous staining and staining of normal kidney are shown in Fig. 1.

### Statistical methods

For statistical analysis, the combined immune score for a cellular compartment was calculated by addition of the intensity score and fraction score, with a resulting scale from 0 to 6 for membrane. For the membranous staining the combined immune score 0–1 was defined as negative tumors and score 2–6 as positive tumors.

Statistical analyses (Kaplan–Meier method, log-rank test) were performed using STATISTICA program (version 2012). A two sided *p* value < 0.05 was defined as statistically significant. The survival statistics was amended with Cox proportional hazards method to establish the influence of any covariates or factors. Ordinary 2 × 2 tables were also used and resulting Chi^2^-tests analyzed. From these tables sensitivity and specificity could be evaluated. As a measure of control logistic models were set up and evaluated both along classical and Bayesian lines.

## Results

### Patients and follow-up

Seventy-seven patients were treated with sunitinib and 59 with sorafenib. Sixteen of the 77 patients and 14 of the 59 patients were excluded from analysis because of early side effects, which lead to the termination of treatment. The 106 remaining patients were treated for a median of 7 months with sunitinib (*n* = 61) or sorafenib (*n* = 45) (range 0.5–40 months). Twelve patients were still on treatment at the end of the follow-up time (Table 3).

**Table 3:** Treatment characteristics for renal cancer patients treated for metastatic disease with sunitinib or sorafenib in the first- or second-line setting

Treatment	Total *n* = 136
Sunitinib, *n* (%)	77 (57)
Sorafenib, *n* (%)	59 (43)
Side effects leading to discontinuation of treatment, *n* (%)	30 (22)
Sunitinib	16
Sorafenib	14
Treated until progression/end of follow-up, *n* (%)	106 (78)
Sunitinib	61
Sorafenib	45
Median PFS, months (range)	7 (0.5–40)
Sunitinib	8
Sorafenib	6
Still under treatment, *n* (%)	12 (11)

There were 77 males and 29 females in this final patient cohort. The median age of diagnosis in this group of patients was 62.5 years (range 33–77). Patients with localized disease at diagnosis (49 patients) were diagnosed with metastases 0–18 years later, median 2 years. Twenty of these 49 patients developed metastatic disease during the first year after diagnosis. Fifty-seven patients had metastatic cancer already at diagnosis.

Median overall survival from the diagnoses of mRCC was 26.5 months (range 1–144 months). At the end of the study there were 17 patients still alive and 89 were deceased (Table 1). The mean length of follow-up available for the surviving patients was 33 months (range 8–84).

### Cubilin expression

**Fig. 2:**
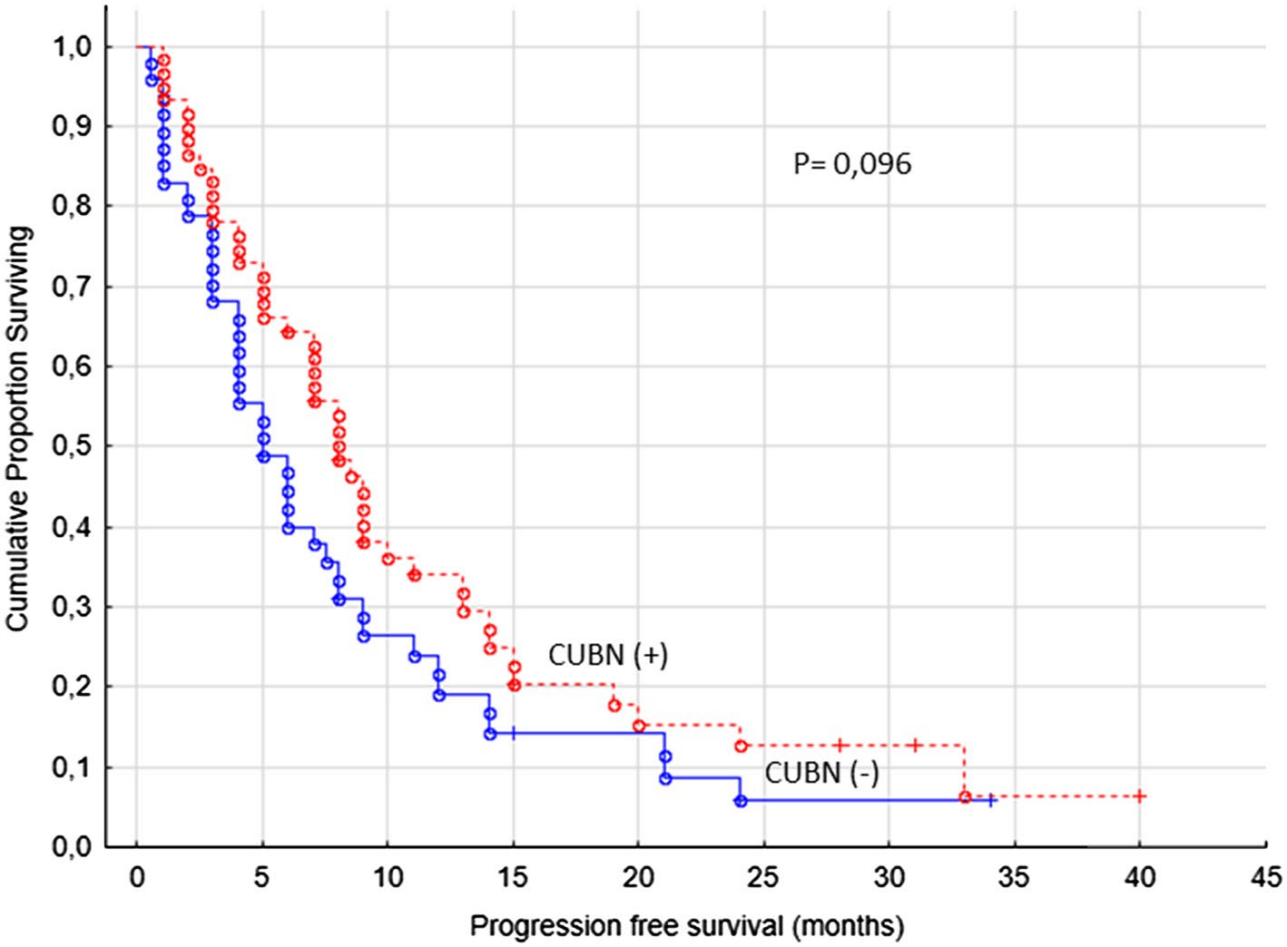
Progression-free survival for renal cancer patients treated for metastatic disease with sunitinib or sorafenib in the first- or second-line setting (*n* = 106), cubilin (−) versus cubilin (+) tumors, cytoplasm

The primary end-point of the study was PFS (defined clinically and/or radiologically) and the second OS in regard with cubilin expression. For cytoplasmic staining we found no correlations with the primary end point of the study (Fig. 2). For the membranous staining, we used the cut-off value for combined staining score described above, which resulted in 53/106 (50%) cubilin positive cases.

**Fig. 3:**
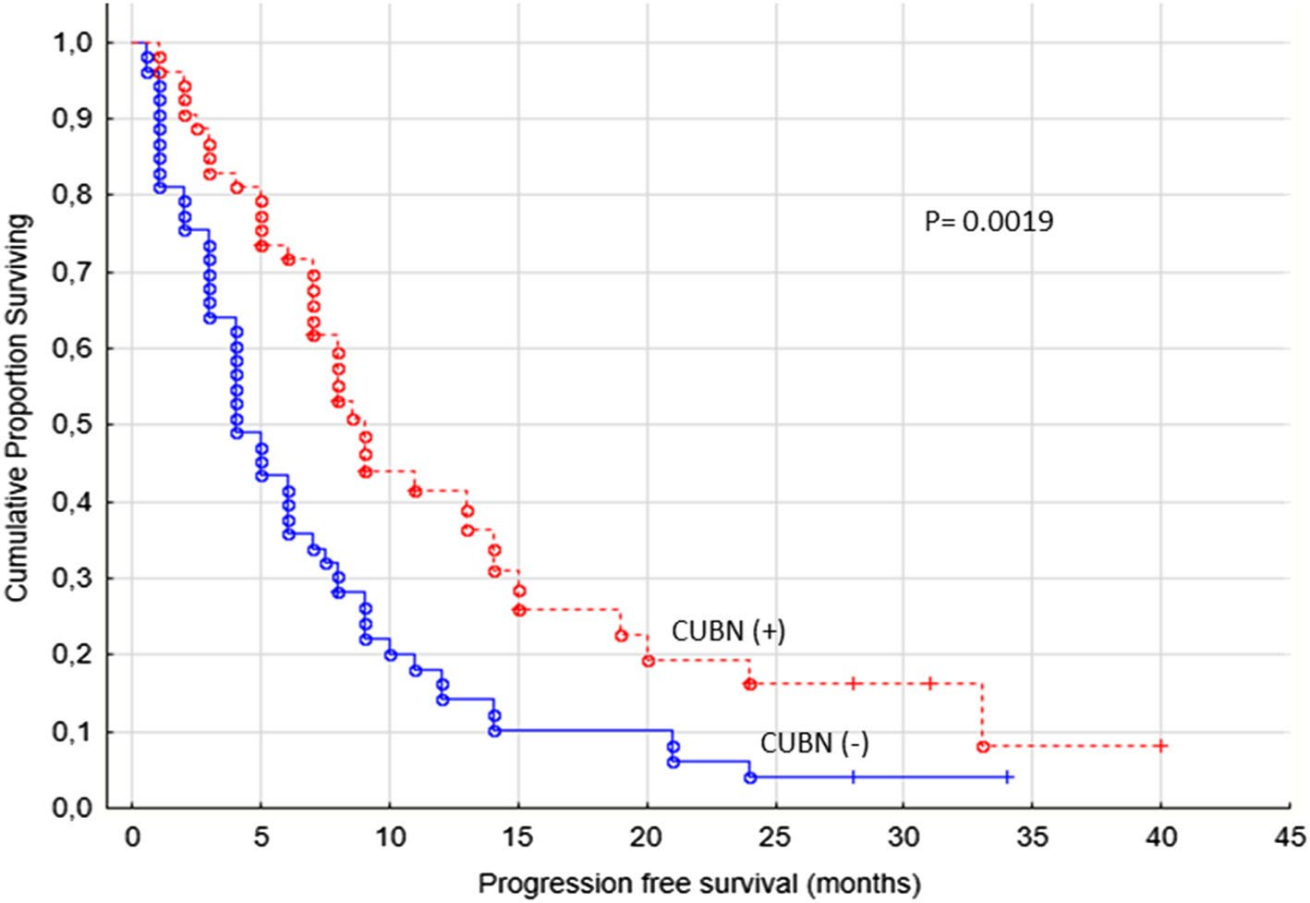
Progression-free survival for renal cancer patients treated for metastatic disease with sunitinib or sorafenib in the first- or second-line setting (*n* = 106), cubilin (−) versus cubilin (+) tumors, membrane

The PFS was significantly better in patients with cubilin expression (*p* = 0.0019, Fig. 3). We observed that patients with cubilin positive tumors were treated with sunitinib or sorafenib in median 8 months (range 1–40 months) compared to cubilin negative patients having a median treatment time of 4 months (range 0.5–34 months).

Patient gender or age at diagnosis of mRCC showed no correlation to the membranous expression of cubilin (*p* values of 0.36 and 0.05, respectively).

Patients with positive cubilin staining had a significantly better OS (*p* = 0.00001, data not shown). The cubilin positive group had a median OS of 36 months (range 7–144 months) while cubilin negative had a median of 15 months (range 1–108 months).

**Fig. 4:**
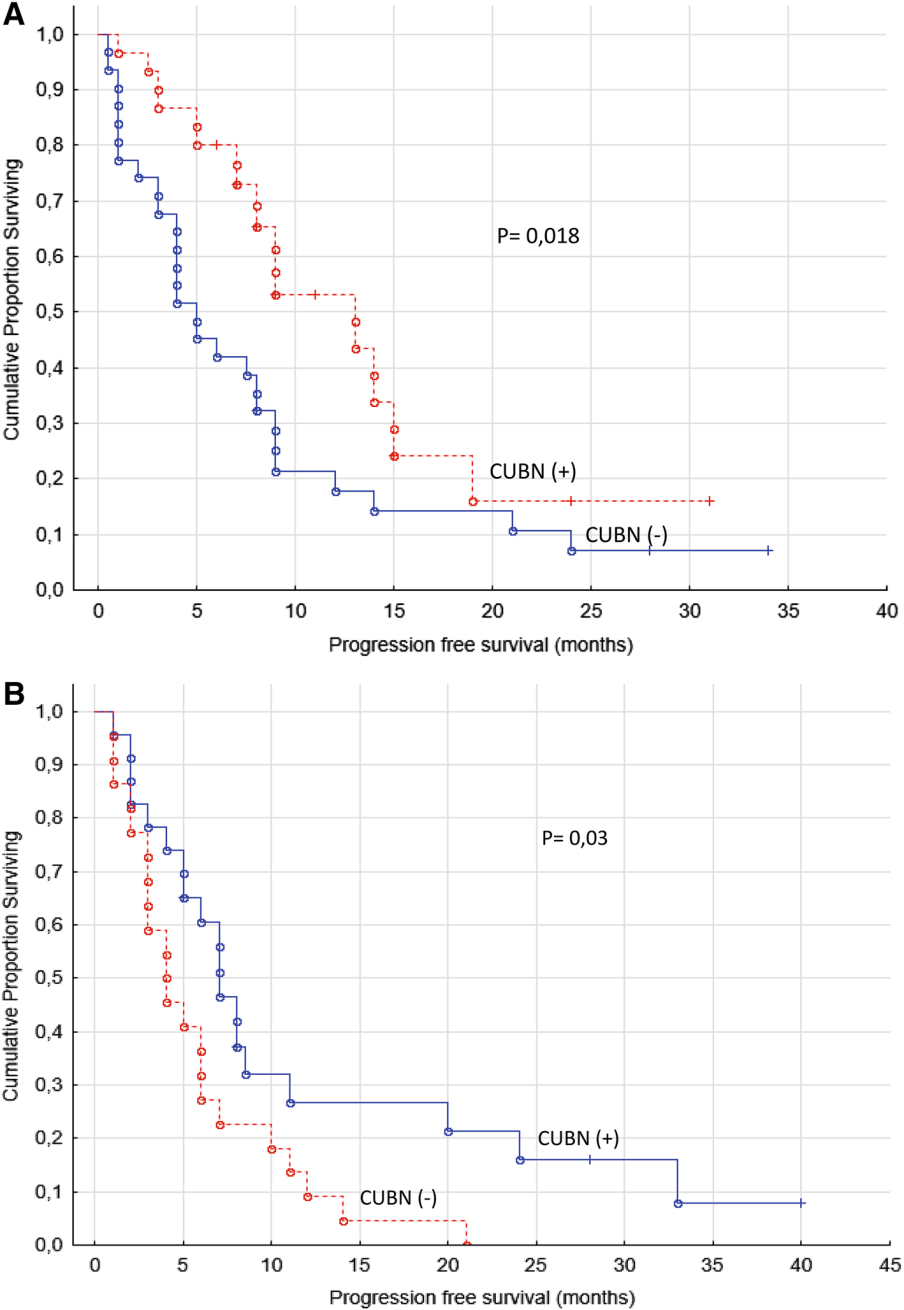
**a** Progression-free survival for renal cancer patients treated for metastatic disease with sunitinib in the first- or second-line setting (*n* = 61). **b** Progression-free survival for renal cancer patients treated for metastatic disease with sorafenib in the first- or second-line setting (*n* = 45)

When PFS was analyzed separately for sunitinib and sorafenib treated groups (two-sample survival analysis) it still resulted in significant differences for positive and negative expression of cubilin (*p* values of 0.02 and 0.03, respectively, log-rank test, Fig. 4).

The fraction of patients with cubilin negative tumors was significantly higher in the non-responding group (*n* = 28) compared to the other patients. PFS was dichotomized along with cubilin expression and the 2 × 2 table gave a Chi^2^ value of 4.85 with a *p* value of 0.028. The sensitivity amounted to 67.9% and the specificity to 56.4%.

## Discussion

The prognosis of mRCC is still very poor although advances in the oncological treatment have been made. Finding molecular targets for RCC has been an area for research in recent years. Sunitinib and sorafenib, two orally administered TKIs, are the first and most used targeted therapies for these patients. An important observation is that some patients who are treated with TKIs benefit much more than the median while others have no gain at all from the treatment (Motzer and A. 2007; Ratain et al. 2006). With a predictive marker responders could be selected for therapy and unnecessary severe toxicity (Di Lorenzo et al. 2011) could be reduced as well as costs of the medication.

Cubilin (gp280) is expressed in the intestinal epithelium and renal proximal tubule epithelium where it functions as a receptor for the complex cobalamine-intrinsic factor. At molecular level, it is a 460 kDa membrane protein with many potential binding sites for various ligands. Together with other proteins such as megalin, cubilin contributes to the internalization of clathrine-coated membrane pits and to the reabsorption of vitamin-carrier proteins, transferrin, hemoglobin etc (Verroust and Christensen 2002). TGF beta downregulates cubilin and is associated with aggressiveness in RCC (Gekle et al. 2003; Sjolund et al. 2011). We have recently demonstrated that cubilin has a prognostic role in RCC patients (Gremel et al. 2017).

In the present study, we used a well-validated antibody to analyze the potential role of tumoral cubilin expression as a predictive marker for sunitinib and sorafenib treatment in mRCC patients.

Both sunitinib and sorafenib prevent tumor angiogenesis, tumor growth and metastasis by inhibiting receptors of VEGF and PDGF (Bergers et al. 2003; Escudier et al. 2012). VEGF- and PDGF-receptors are overexpressed in clear cell RCC due to inactivation of the tumor-suppressor gene von Hippel-Lindau (VHL) in at least 60% of the cases (van der Veldt et al. 2008).

Markers for early evaluation of response are established for sorafenib as well as sunitinib treatment. Hypertension related to sunitinib or sorafenib treatment in patients with mRCC is associated with a better response and prolonged OS (Rixe et al. 2007; Szmit et al. 2012a, b). In a retrospective analysis of over 500 patients those who developed hypertension during sunitinib treatment had significantly longer PFS and OS compared to patients not developing hypertension (Rini et al. 2011). The median time to initiation of antihypertensive treatment was 28 days calculated from the start of the sunitinib treatment (range 10–80 days) (Bono et al. 2011). Hand-foot skin reaction (HFSR) is one of the common adverse events in patients treated with sorafenib and develop early in the course of the treatment, the majority during the first cycle (Hutson et al. 2010). In a cohort of over 700 patients treated with sunitinib or sorafenib, the presence of skin toxicity was associated with improved OS and PFS in the sunitinib subgroup. (Poprach et al. 2012).

Just few potential predictive biomarkers for sorafenib have been studied. In a study with cfDNA, levels in patients with mRCC (*n* = 18) were significantly higher than those in healthy controls (*n* = 10). Baseline levels of plasma cfDNA were not associated with response to sorafenib treatment but a significantly lower level, measured from week 8 to 24 weeks, was found in patients with remission or stable disease than in those with progression (Feng et al. 2013).

The majority of the previous predictive marker studies in mRCC patients have focused on serum biomarkers for sunitinib treatment. Studying baseline levels of TNF-α and MMP-9 in 21 sunitinib-treated patients significantly increased levels in non-responders were measured (Perez-Gracia et al. 2009). Levels of these two proteins, which promote cancer development (Bergers et al. 2000; Harrison et al. 2007), were significantly associated with a reduced time to progression (TTP) and OS. In another study serum-VEGF and -NGAL were evaluated. NGAL is strongly expressed in inflammatory, pre-tumoral and neoplastic lesions and tightly correlated with MMP-9. High baseline levels predicted a higher relative risk of progression in mRCC patients (*n* = 85) treated with sunitinib (Porta et al. 2010).

In a TMA-study like ours, with substantially fewer mRCC patients (*n* = 42), potential predictive markers for response to sunitinib treatment were investigated. Hypoxia-inducible factor 1α (HIF-1α), CA9, CD31, pVEGFR1, VEGFR1 and VEGFR2, pPDGFRα and -β and Ki67 were all associated with sunitinib response. In addition, a high HIF-1α expression was positively correlated to a longer PFS and a low PDGFRα score to a longer OS. Furthermore, patients with a low CA9 score (*n* = 19) had a median OS of 22 months compared to patients with a high CA9 score (*n* = 9) with a median OS of 48 months (Dornbusch et al. 2013). In another study, tumor expression of programmed death-1 ligand (PD-L1) was analyzed in advanced RCC patients receiving VEGF-targeted therapy (pazobanib or sunitinib). Both PFS and OS were significantly shorter in patients with increased tumor cell PD-L1 or PD-L1 plus tumor CD8-positive T cell counts (Choueiri et al. 2015).

In our study, we found that patients with membranous cubilin expression in their primary tumors experienced a greater clinical benefit from sunitinib and sorafenib treatment in terms of a doubled PFS. Since different tyrosine kinase inhibitors function differently to some extent, it is possible that they require separate predictive markers. However, when analyzing sunitinib and sorafenib treated patients separately the difference in PFS remained significant. These findings indicate that membranous cubilin expression is a predictive factor for both sunitinib and sorafenib treatment. Whether cubilin’s predictive value extends to all TKIs remains to be investigated.

In addition to PFS, the OS was also significantly longer in patients with cubilin positive tumors compared to the patients with cubilin negative tumors. A plausible explanation is that the gain in PFS is translated into a longer OS. However, several of the patients were treated with other therapeutic agents, which could contribute to the difference in OS observed between the groups. Moreover, cubilin expression might predict survival independent of treatment (Gremel et al. 2017).

Patients are usually evaluated both clinically and radiologically after two months of treatment. In a subanalysis we focused on patients treated ≤3 months with sunitinib or sorafenib and regarded these patients as non-responders. Our study showed that a significantly higher fraction of patients in the non-responding group had cubilin negative tumors. More studies are needed to explore whether it is possible to better select the minor group of patients with no benefit at all. One possible strategy would be to combine cubilin membranous expression with another putative predictive marker.

This study has some limitations. Due to its retrospective design, known serum prognostic markers (lactate dehydrogenase, hemoglobin, calcium) could for many patients not be recalled. Therefore, we were unable to assess whether cubilin has any prognostic value besides from being a predictive marker. Furthermore, the tumor response was not on a regular basis evaluated according to the Response Evaluation Criteria In Solid Tumors (RECIST) (Therasse et al. 2000).

We show for the first time that cubilin tumoral expression is of predictive value for treatment of mRCC patients, a strong association with PFS was observed. In addition, a significantly higher fraction of patients in the non-responding group had cubilin negative tumors. Further studies are needed to investigate whether cubilin is a predictive marker for all TKIs. Another aim for future research is to more accurately delineate the non-responding group since a better selection is warranted before starting to use cubilin as a predictive marker in the clinic.
